# The Effects of Combined Exercise Training (Resistance-Aerobic) on Serum Kinesin and Physical Function in Type 2 Diabetes Patients with Diabetic Peripheral Neuropathy (Randomized Controlled Trials)

**DOI:** 10.1155/2020/6978128

**Published:** 2020-03-06

**Authors:** Seyedeh Hoda Seyedizadeh, Sadegh Cheragh-Birjandi, Mohammad Reza Hamedi Nia

**Affiliations:** ^1^Department of Sport Science, Islamic Azad University, Bojnourd Branch, Bojnourd, Iran; ^2^Faculty of Sport Sciences, Hakim Sabzevari University, Sabzevar, Iran

## Abstract

Diabetic peripheral neuropathy is one of the most common chronic complications of diabetics which causes nerve damage and muscle strength decrease in patients. This in turn results in imbalance leading to the diabetic patients' daily activity disparity. The present investigation was conducted to specifically study the effects of combined training (resistance-aerobic) on serum kinesin-1 and physical function in type 2 diabetes patients with diabetic peripheral neuropathy. 24 diabetic neuropathic females were randomly to be selected out and divided into two experimental and control groups. The experimental group received resistance-aerobic training for 3 sessions during eight weeks. The exercise training included resistance exercises with 2-3 sets, 6-7 exercise stations, 8-12 repetitions (reps), and 3-5 minutes of rest in between the exercises, and the aerobic exercises contained 50-65% of heart rate reserve (HRR) for 3 minutes with 30 seconds of rest interval between sets and 5-10 repetitions. Results show that the serum kinesin-1 level and aerobic endurance declined after eight weeks of combined (resistance-aerobic) exercise training, but this decrease was not significant. The upper body strength increased but it was not significant, while the lower body showed a significant strength increase. With regard to the progressive nature of diabetic peripheral neuropathy, it seems that even the little changes resulting from the combined exercise training can be useful. Nevertheless, more research is required in this area.

## 1. Introduction

The diabetic peripheral neuropathy is one of the most common complications resulting from diabetes. This complication emerges with such symptoms in patients as severe pain, increase in injury or wound danger and amputation, sense loss or decrease, tingling sensation in hands or feet, menopausal hot flashes, instability increase and balance, and performance disorder [1]. The factors affecting the diabetic peripheral neuropathic pathogens are the hyperglycemia, nitrogen and oxidative stress, mitochondrial function disorder, polyol pathway activation, advanced glycation end product (AGE) increase, and irritation symptom increase [2, 3] which result in destruction of neurons and axons in sensory and motor nerves. Nerve damage in the diabetic peripheral neuropathy follows a specific destructive pattern in such a way that first sensory nerves and then motor ones are involved, and this involvement appears as dying-back. It means that the lower axons die before the complete destruction of the nervous system (nerve tree) [4]. It seems that the dying-back pattern results from a disorder or progression in the production of proteins and necessary matters needed for axons. This pattern is more seen in longer axons [4].

The large distance of axons from the cellular body causes the materials required for its preserving and functioning to get transported more quickly by the axonal transport system. This system includes microtubules and motor kinesin and dynein proteins [5, 6]. Kinesin-1 with the energy resulting from ATP (Adenosine triphosphate) hydrolysis moves over the microtubules and is responsible for the movement of many of cell shipments. The connection of cell shipments to kinesin-1 is established through two kinesin light chains (KLC) [7]. KLC1 is responsible for connecting intercellular shipments such as cellular mitochondria and proteins [8] and plays a major role in axonal (nerve) transport [9].

The disorder in the axonal transport system results in the destruction of motor units and neurogenic muscular atrophy [10]. It seems that with a person's chronic affliction to diabetic peripheral neuropathy and its major increase, the motor axons are more subject to destruction and at last results in more muscular tissue death [11]. Neurogenic muscular atrophy declines patients' muscular strength, speed, and endurance and is along with neural fatigue leading to the reduction of their potential performance [12, 13]. In fact, it seems that losing muscular strength in the lower body is the major cause of walking imbalance and disorders in the diabetic peripheral neuropathic patients. This increases the danger of falling down [13].

The purpose of treating the diabetic peripheral neuropathy is to prevent the advancement of neuropathic symptoms and neural function disorders and its decadence, but more importantly is to expand the rebuilding of immature fiber degeneration [14]. Although regular physical exercise most probably cannot completely divert the peripheral neuropathic symptoms, it can prevent from more muscular strength loss and flexibility decline [15]; moreover, it can relieve the diabetic peripheral neuropathic pain [16] and improves the neural function [15, 17]. Studies indicate that combined resistance and aerobic exercise training remarkably reduces neuropathic symptoms and pain, increases muscular fiber [15], and improves walking strength and balance [18, 19]. It also improves skin neural density [19] and reduces general and physical fatigue [20]. Based on our knowledge, the effect of combined resistance and aerobic exercise training on serum kinesin in the diabetic peripheral neuropathic patients has not been investigated in studies so far, the present study was conducted to show whether it can affect the serum KLC1 variations, aerobic resistance, and upper and lower body strength.

## 2. Methods

This trial designed a parallel group including allocation ratio 1 : 1 with a pretest and posttest, carried out in Sabzevar, Iran. After calling for subjects' entering the research project, 24 females were randomly (Simple) selected and then randomly divided into two equal groups: control and experimental (*N* = 12). For randomization, we use flipping a coin for the two treatment groups (control versus experimental); the side of the coin (i.e., heads-control and tails-experimental) determines the assignment of each subject. The approval of diabetic peripheral neuropathy (by applying the Michigan Questionnaire and Monofilament 10-Point Test), the age range of 45-65 years, the subjects' ability to do resistance and aerobic exercises, and the females' menopause were the inclusion criteria for this study. The exclusion criteria for the study were such problems as amputation, sole injury, severe retinopathy, dialysis and neuropathy, upper body neuropathy or arthritis that could help reduce and/or limit the pain, the existence of imbalance factors except for neuropathy, subjects' not attending the initial and final tests and sample taking, and absenting from the exercise for more than 3 sessions. Due to their having some of the criteria, two of the subjects in the experimental group had to leave the study before it was terminated. Also, patients were asked to complete forms “PAR-Q+” (Annex 1) and “Informed Consent Form Template” (Annex 2) before the protocol started.

Note: this research was approved by the Ethics Committee of Sabzevar University of Medical Sciences with number IR.MEDSAB.REC.1396.3.

### 2.1. The Training Protocol

The training program was implemented for 3 sessions per week for 8 weeks. In the beginning of each session of exercise, the subjects warmed up for 15 minutes and then did the resistance exercises. Having done the resistance exercises, they took a rest of 3-5 minutes and started doing aerobic exercises and cooled off after they exercised for 10-15 minutes. Warm up exercises contained walking for 5 minutes and doing the training protocol strength exercises without any load for 10 minutes. Cooling down consisted of 5 minutes for walking and 5-10 minutes for stretching exercises.

### 2.2. Resistance Training

The resistance training included chest press, wide-grip lat pulldown, barbell curl, lying triceps press, leg extension, lying leg curls, sit-up, and push-up using bodybuilding equipment. The movements started with the stations that involved bigger muscles, the movements such as leg extension, and then moved to and finished in the stations that activated the smaller muscles like barbell curl. The training intensity was managed in such a way that the subjects could do each movement for 8-12 times repetitively; in case a subject managed to carry out each exercise movement for 12 times in two sessions in a row, the used weight increased in the next session. The exercises were carried out in two sets for the first four weeks and after the fifth week all the movements were followed in three sets to the end of the program. The number of stations was 6 until the seventh week and in the eighth week it was increased to 7. The number of repetitions in the whole period of the exercise was fixed and unchangeable and it was 8-12 reps. The rest time between the sets was one minute and between the stations it was two minutes.

### 2.3. Interval Aerobic Training

The aerobic training included the interval running with 3-minute repetitions and a rest time of 30 seconds. The intensity of aerobic training started with 50% of heart rate reserve (HRR) [21] in the first week and was increased to 65% in the eighth week. The number of repetitions also started with 5 in the first week and was terminated with 10 in the eighth week.

### 2.4. Functional Tests

To test the subjects' aerobic resistance, upper body (trunk) and lower body strength, the 6-minute walking test, the 30-second bicep curl test, and Rikli and Jones Chair Stand Test were administered [22].

### 2.5. Blood Sample Taking

The blood samples (5_cc_) were taken from the patients' veins 24 hours before and 48 hours after the training program after a 12-hour night fasting. The samples were poured in test tubes without anticoagulant agent. To prepare the blood serum, the samples were kept in room temperature for 10 minutes, their clot being removed, and then were centrifuged at 3000 rpm for 20 minutes. The samples were frozen at -20°C. The KLC1 serum level was measured by using the human ELIZA kit with a sensitivity level of 2.44 ng/ml and CV of 5% (EASTBIOPHARM-China).

### 2.6. Statistical Analysis

To analyze the data, the SPSS-23 was used. To calculate the standard deviation and the mean, the descriptive statistics was applied. After getting assured about the normality of the data by using the Kolmogorov-Smirnov Normality Test and analysis of homogeneity variances using the Levene's Test, repeated measures ANOVA was used for the analysis of the data.

## 3. Result

The data analysis in Table 1 shows that the anthropometric data of the training and control groups did not significantly differ in the pretest (*P* < 0.05).

The results indicate that the KLC1 serum levels did not change significantly after eight weeks in both groups (*P* > 0.05); furthermore, there were no significant differences between the two groups in their serum levels of KLC1 (*P* > 0.05), but effect size data shows that training could moderately effect on KLC1 levels. The aerobic endurance difference both in the treatment group and in the control group after eight weeks was not significant (*P* > 0.05); however, the comparison between the two levels of aerobic endurance of both groups showed that after eight weeks the aerobic endurance in the control group significantly decreased (*P* = 0.008), and effect size data shows that training could moderately effect on aerobic endurance. The upper body strength level both within and between groups did not significantly vary (*P* > 0.05) after eight weeks; nevertheless, the lower body strength in training group significantly increased after eight weeks (*P* = 0.001), and data shows that the effect size of training on lower body strength is strong.

## 4. Discussion

The diabetic peripheral neuropathy causes structural variations in the peripheral neurons [23], and neural transport system that functions with kinesin and dynein motor proteins gets functionally impaired [6]. Because of the destruction of neurons, proteins and other materials of the damaged neurons enter the blood and are disposed [24]. Various studies indicate that resistance and aerobic training helps purge the diabetic peripheral neuropathic complications [15, 25, 26]. However, the findings of the present study proved that the combined resistance-aerobic training did not show a significant difference in the KLC1 serum level after eight weeks [27]. To the best of our knowledge, the present study was the first research work that investigated the KLC1 variations in the subjects afflicted with diabetic neuropathy. In some similar works carried out on animals, the kinesin variation in their nervous system was investigated. As Golbar et al. [28] and Rahmati et al. (2013) indicated, the endurance training significantly increased the kinesin level and, in turn, its mRNA in the sciatic nerves of the healthy rats' treatment group, compared with that of the healthy rats control group; however, in the diabetic treatment group the kinesin and, in turn, its mRNA levels declined thanks to the training, compared with those of the diabetic control group. These studies indicated that the KLC1 level in the diabetic control group is more than that of the training-diabetic group and healthy control and training groups where the findings are in line with the findings of the study conducted by Baptista et al. [29] that showed an increase expression of Hippocampus KLC-1 in rats [29]. The constant increase of kinesin-1 content in the sciatic nerves of the diabetic neuropathic rats is probably because of dying-back of the neurons in neuropathy and the activation of compensating mechanism. However, in response to the logical and effective exercise activities, neurons get activated and balance this content helping it reach a normal level [28, 29]. Further, in line with these findings, some other research works are suggestive of the same decrease in the level of kinesin or disorder in that content. For example, in rats afflicted with parkinsonism [30, 31] and in patients afflicted with ALS [32] and Alzheimer's disease, the content of kinesin reduces [33]; however, the KLC1 level in patients with breast cancer was witnessed to be remarkably increasing [34]. It is worth mentioning that in the present study, it was observed that the dose of medicine used by the subjects varied in such a way that the drug dose in the control group increased, while in the training group it decreased. This can justify the decrease of KLC1 serum level in the control group and the insignificance of the training group's KLC1 serum level variation, and this is consistent with the effect size findings in Table 2. The results of the present study showed that aerobic endurance reduced in both groups, but the reduction level in the training group was less than that of the control group. It seems that the training program had some benefit on aerobic endurance. It seems that the findings of the present research are in line with those of Kluding et al. [15] where they found that regular physical exercise could not reverse the peripheral neuropathic symptoms but could prevent the progression or advancement of the disease [15]. The findings of the current work also showed that combined resistance-aerobic training could not completely stop the diabetic peripheral neuropathic patients' walking disorders, but they could reduce the progression or advancement pace up to 1/5. In the investigation of other studies, it can be observed that aerobic training [35] and combined (aerobic-resistance) ones [36] increased the duration of the diabetic patients' walking where the findings differ from those of the present study. This difference may be due to the subjects' individual differences in that the subjects of these two researches were not afflicted with diabetic neuropathy. Two other research works also investigated the effects of aerobic training on the diabetic neuropathic patients whose results were contradictory. Another point in focus is that while the findings of the study conducted by Morrison et al. [37] showed a significant improvement in the patients' walking, response time, balance indices, and dynamic position, those of the study carried out by Kruse et al. (2010) showed that the combined resistance-aerobic training brought about no improvement in the balance and endurance of the patients' lower body [37]. This incongruity might be due to the intensity and type of the exercise protocol.

Diabetic neuropathy results in disorders in the nervous transport system and finally in muscular neurogenic atrophy [10]. Muscular atrophy causes the muscular strength, speed, and endurance to decline [12, 13]. In the present study, the trunk strength increase in the treatment group was insignificant, but in the control group it declined. The decline in the control group subjects' muscular strength of their trunk might be due to the progressing neuropathy; however, this was not the same as in the treatment group: the combined training not only prevented from the progression of the disease, but also helped increase the patients' trunk muscular strength compared with its status in the pretest. In line with the findings of the present study, some other research works showed a congruence of their findings. They indicate that the aerobic-resistance exercises increase muscular strength and endurance [38–41]. The results of the present study also showed that lower body strength significantly increased in the combined training group but decreased in the control group. These findings, along with those of the other studies, show that aerobic and resistance training can potentially enhance the type 2 diabetic patients' muscular strength [42–44] and that of the patients afflicted with diabetic neuropathy [45–47]. Here, one question is raised: while experiencing a significant increase in the lower body strength, why cannot we observe any improvement in aerobic endurance? To answer this question, it can be said that in order to walk and keep balanced, an individual, in addition to his muscular strength [48], needs to enjoy such factors as quick response time, spatial perception, body sense feedbacks, visual information [49], cognitive function, and behavioral-cognitive understanding factors [50]. Any disorder in any of them results in losing the balance and falling down [51]. The age attribute increase might have had a significant effect on each issue stated above and might have caused the balance loss.

## 5. Conclusion

In regard to the progressive nature of diabetic peripheral neuropathy and the limited time of the combined aerobic and resistance treatment, it seems that the little variations having occurred in KLC-1 serum, aerobic endurance, and trunk strength can be positively taken and evaluated, and in spite of the fact that these variations are not statistically significant, it can be predicted that probably longer periods of these trainings can bring about effective results. Regarding the fact that the lower body strength increase is significant, it can be concluded that the combined aerobic-resistance training has had a significant effect on the lower body strengthening factors, and it is felt that more research is required to be conducted in this field.

## Figures and Tables

**Table 1:** Subjects' anthropometric characteristics.

Attributes	Group			*P*
	Test	Control (*n* = 10)	Resistance-aerobic treatment group (*n* = 12)	
Age (years)	—	60.81 ± 5.17	56.69 ± 4.13	—
Height (cm)	—	158 ± 5	155 ± 4	—
Weight (kg)	Pretest	70 ± 10.81	73.31 ± 14.99	0.58
	Pretest	70.48 ± 11.08	72.18 ± 14.73	
BMI (kg per m^2^)	Pretest	28.05 ± 4.53	29.99 ± 5.06	0.38
	Pretest	28.24 ± 4.62	29.88 ± 5.05	
Fat percent	Pretest	39.84 ± 3.42	41.58 ± 3.39	
				0.46
	Pretest	37.96 ± 4.50	39.88 ± 3.46	
WHR	Pretest	0.90 ± 0.04	0.86 ± 0.03	0.09
	Pretest	0.88 ± 0.03	0.86 ± 0.02	

BMI: body mass index; WHR: waist-to-hip ratio. *P* < 0.05 is significant. *P* value is for ANOVA of pretest data.

**Table 2:** The results of the repeated measures ANOVA.

Variable	Group	Mean and SD		Intragroup variation		Partial eta squared	Observed power	Intergroup variation		Partial eta squared	Observed power
		Pretest	Posttest	*F*	*P* ^∗^			*F*	*P* ^∗∗^		
KLC1 (ngr)	Control	363.28 ± 60.95	343.22 ± 36.26	0.94	0.36	0.007	0.065	0.05	0.83	0.154	0.442
	Treatment	367.29 ± 31.87	337.29 ± 53.75	3.22	0.1						
Aerobic resistance (m)	Control	405.40 ± 51.43	337.20 ± 58.12	3.76	0.10	0.337	0.791	11.70	0.008^∗^	0.292	0.704
	Treatment	445.75 ± 58.80	432.16 ± 34.92	1.3	0.28						
Trunk strength (numbers)	Control	17.20 ± 4.67	16.20 ± 2.20	0.59	0.46	0.109	0.320	2	0.19	0.004	0.057
	Treatment	18.58 ± 3.23	19.17 ± 4.43	2.04	0.54						
Lower body strength (numbers)	Control	11.50 ± 0.85	10.30 ± 0.95	36	0.001^∗^	0.146	0.421	10.06	0.01^∗^	0.72	0.222
	Treatment	11.25 ± 2	13.42 ± 3.09	10.05	0.01^∗^						

KLC1: kinesin light chain 1; *P*^∗^: intragroup significant level; *P*^∗∗^: intergroup significant level.

## Data Availability

The [Anthropometric Characteristics, KLC1, Aerobic Resistance, Trunk Strength, Lowerbody Strength] data used to support the findings of this study are available from the corresponding author upon request.

## Supplementary Materials

Supplementary Materials Physical Activity Readiness Questionnaire (PAR-Q+) is the main screening tool for physical activity/participation in sport. The PAR-Q+ is completed by people who want to do a “very physical” fitness assessment or exercise. When a participant gives a positive response to PAR-Q+, they are consulted by a physician to discharge activity in unlimited or limited physical activity.
