# Supplementary material for: The Effects of Combined Exercise Training (Resistance-Aerobic) on Serum Kinesin and Physical Function in Type 2 Diabetes Patients with Diabetic Peripheral Neuropathy (Randomized Controlled Trials)
Source: J Diabetes Res. 2020 Mar 6;2020:6978128. doi: 10.1155/2020/6978128 (PMC7085367; doi:10.1155/2020/6978128)
Supplement: Supplementary Materials — Physical Activity Readiness Questionnaire (PAR-Q+) is the main screening tool for physical activity/participation in sport. The PAR-Q+ is completed by people who want to do a “very physical” fitness assessment or exercise. When a participant gives a positive response to PAR-Q+, they are consulted by a physician to discharge activity in unlimited or limited physical activity. [file 6978128.f1.pdf]

PAR-Q $^{+}$

Physical Activity Readiness Questionnaire (PAR-Q^{+}) is the main screening tool for physical activity / participation in sport. The PAR-Q^{+} is completed by people who want to do a "very physical" fitness assessment or exercise. When a participant gives a positive response to PAR-Q^{+}, they are consulted by a physician to discharge activity in unlimited or limited physical activity.

Although, all participants were examined by a physician at the beginning of this research work, we asked them to complete PAR-Q^{+} form to we were made sure everything is ok.

PAR-Q^{+} (CSEP approved Sept 12 2011 version)

The Physical Activity Readiness Questionnaire for Everyone

Regular physical activity is fun and healthy, and more people should become more physically active every day of the week. Being more physically active is very safe for MOST people. This questionnaire will tell you whether it is necessary for you to seek further advice from your doctor OR a qualified exercise professional before becoming more physically active.

SECTION 1 - GENERAL HEALTH

	Please read the 7 questions below carefully and answer each one honestly: check YES or NO.	YES	NO
1.	Has your doctor ever said that you have a heart condition OR high blood pressure?	☐	☐
2.	Do you feel pain in your chest at rest, during your daily activities of living, OR when you do physical activity?	☐	☐
3.	Do you lose balance because of dizziness OR have you lost consciousness in the last 12 months? Please answer NO if your dizziness was associated with over-breathing (including during vigorous exercise).	☐	☐
4.	Have you ever been diagnosed with another chronic medical condition (other than heart disease or high blood pressure)?	☐	☐
5.	Are you currently taking prescribed medications for a chronic medical condition?	☐	☐
6.	Do you have a bone or joint problem that could be made worse by becoming more physically active? Please answer NO if you had a joint problem in the past, but it does not limit your current ability to be physically active. For example, knee, ankle, shoulder or other.	☐	☐
7.	Has your doctor ever said that you should only do medically supervised physical activity?	☐	☐

If you answered NO to all of the questions above, you are cleared for physical activity.

Go to Section 3 to sign the form. You do not need to complete Section 2.

›› Start becoming much more physically active – start slowly and build up gradually.
›› Follow the Canadian Physical Activity Guidelines for your age (www.csep.ca/guidelines).
›› You may take part in a health and fitness appraisal.
›› If you have any further questions, contact a qualified exercise professional such as a CSEP Certified Exercise Physiologist® (CSEP-CEP) or CSEP Certified Personal Trainer® (CSEP-CPT).
›› If you are over the age of 45 yrs. and NOT accustomed to regular vigorous physical activity, please consult a qualified exercise professional (CSEP-CEP) before engaging in maximal effort exercise.

If you answered YES to one or more of the questions above, please GO TO SECTION 2.

Delay becoming more active if:

›› You are not feeling well because of a temporary illness such as a cold or fever – wait until you feel better
›› You are pregnant – talk to your health care practitioner, your physician, a qualified exercise professional, and/or complete the PARmed-X for Pregnancy before becoming more physically active OR
›› Your health changes – please answer the questions on Section 2 of this document and/or talk to your doctor or qualified exercise professional (CSEP-CEP or CSEP-CPT) before continuing with any physical activity programme.

SECTION 2 - CHRONIC MEDICAL CONDITIONS

	Please read the questions below carefully and answer each one honestly: check YES or NO.	YES	NO
1.	Do you have Arthritis, Osteoporosis, or Back Problems?	☐ If yes, answer questions 1a-1c	☐ If no, go to question 2
1a.	Do you have difficulty controlling your condition with medications or other physician-prescribed therapies? (Answer NO if you are not currently taking medications or other treatments)	☐	☐
1b.	Do you have joint problems causing pain, a recent fracture or fracture caused by osteoporosis or cancer, displaced vertebra (e.g., spondylolisthesis), and/ or spondylolysis/pars defect (a crack in the bony ring on the back of the spinal column)?	☐	☐
1c.	Have you had steroid injections or taken steroid tablets regularly for more than 3 months?	☐	☐
2.	Do you have Cancer of any kind?	☐ If yes, answer questions 2a-2b	☐ If no, go to question 3
2a.	Does your cancer diagnosis include any of the following types: lung/bronchogenic, multiple myeloma (cancer of plasma cells), head, and neck?	☐	☐
2b.	Are you currently receiving cancer therapy (such as chemotherapy or radiotherapy)?	☐	☐
3.	Do you have Heart Disease or Cardiovascular Disease? This includes Coronary Artery Disease, High Blood Pressure, Heart Failure, Diagnosed Abnormality of Heart Rhythm	☐ If yes, answer questions 3a-3e	☐ If no, go to question 4
3a.	Do you have difficulty controlling your condition with medications or other physician-prescribed therapies? (Answer NO if you are not currently taking medications or other treatments)	☐	☐
3b.	Do you have an irregular heart beat that requires medical management? (e.g. atrial fibrillation, premature ventricular contraction)	☐	☐
3c.	Do you have chronic heart failure?	☐	☐
3d.	Do you have a resting blood pressure equal to or greater than 160/90 mmHg with or without medication? (Answer YES if you do not know your resting blood pressure)	☐	☐
3e.	Do you have diagnosed coronary artery (cardiovascular) disease and have not participated in regular physical activity in the last 2 months?	☐	☐
4.	Do you have any Metabolic Conditions? This includes Type 1 Diabetes, Type 2 Diabetes, Pre-Diabetes	☐ If yes, answer questions 4a-4c	☐ If no, go to question 5
4a.	Is your blood sugar often above 13.0 mmol/L? (Answer YES if you are not sure)	☐	☐
4b.	Do you have any signs or symptoms of diabetes complications such as heart or vascular disease and/or complications affecting your eyes, kidneys, and the sensation in your toes and feet?	☐	☐
4c.	Do you have other metabolic conditions (such as thyroid disorders, pregnancy-related diabetes, chronic kidney disease, liver problems)?	☐	☐
5.	Do you have any Mental Health Problems or Learning Difficulties? This includes Alzheimer’s, Dementia, Depression, Anxiety Disorder, Eating Disorder, Psychotic Disorder, Intellectual Disability, Down Syndrome)	☐ If yes, answer questions 5a-5b	☐ If no, go to question 6
5a.	Do you have difficulty controlling your condition with medications or other physician-prescribed therapies? (Answer NO if you are not currently taking medications or other treatments)	☐	☐
5b.	Do you also have back problems affecting nerves or muscles?	☐	☐

CSEP | SCPE THE GOLD STANDARD IN EXERCISE SCIENCE AND PERSONAL TRAINING
COPYRIGHT © 2012
CSEP approved Sept 12 2011 version

		Please read the questions below carefully and answer each one honestly: check YES or NO.	YES	NO
6.		Do you have a Respiratory Disease?<br>This includes Chronic Obstructive Pulmonary Disease, Asthma, Pulmonary High Blood Pressure	☐ If yes, answer questions 6a-6d	☐ If no, go to question 7
	6a.	Do you have difficulty controlling your condition with medications or other physician-prescribed therapies?<br>(Answer NO if you are not currently taking medications or other treatments)	☐	☐
	6b.	Has your doctor ever said your blood oxygen level is low at rest or during exercise and/or that you require supplemental oxygen therapy?	☐	☐
	6c.	If asthmatic, do you currently have symptoms of chest tightness, wheezing, laboured breathing, consistent cough (more than 2 days/week), or have you used your rescue medication more than twice in the last week?	☐	☐
	6d.	Has your doctor ever said you have high blood pressure in the blood vessels of your lungs?	☐	☐
7.		Do you have a Spinal Cord Injury? This includes Tetraplegia and Paraplegia	☐ If yes, answer questions 7a-7c	☐ If no, go to question 8
	7a.	Do you have difficulty controlling your condition with medications or other physician-prescribed therapies?<br>(Answer NO if you are not currently taking medications or other treatments)	☐	☐
	7b.	Do you commonly exhibit low resting blood pressure significant enough to cause dizziness, light-headedness, and/or fainting?	☐	☐
	7c.	Has your physician indicated that you exhibit sudden bouts of high blood pressure (known as Autonomic Dysreflexia)?	☐	☐
8.		Have you had a Stroke?<br>This includes Transient Ischemic Attack (TIA) or Cerebrovascular Event	☐ If yes, answer questions 8a-c	☐ If no, go to question 9
	8a.	Do you have difficulty controlling your condition with medications or other physician-prescribed therapies?<br>(Answer NO if you are not currently taking medications or other treatments)	☐	☐
	8b.	Do you have any impairment in walking or mobility?	☐	☐
	8c.	Have you experienced a stroke or impairment in nerves or muscles in the past 6 months?	☐	☐
9.		Do you have any other medical condition not listed above or do you live with two chronic conditions?	☐ If yes, answer questions 9a-c	☐ If no, read the advice on page 4
	9a.	Have you experienced a blackout, fainted, or lost consciousness as a result of a head injury within the last 12 months OR have you had a diagnosed concussion within the last 12 months?	☐	☐
	9b.	Do you have a medical condition that is not listed<br>(such as epilepsy, neurological conditions, kidney problems)?	☐	☐
	9c.	Do you currently live with two chronic conditions?	☐	☐

Please proceed to Page 4 for recommendations for your current medical condition and sign this document.

PAR-Q+

If you answered NO to all of the follow-up questions about your medical condition, you are ready to become more physically active:

›› It is advised that you consult a qualified exercise professional (e.g., a CSEP-CEP or CSEP-CPT) to help you develop a safe and effective physical activity plan to meet your health needs.

›› You are encouraged to start slowly and build up gradually – 20-60 min. of low- to moderate-intensity exercise, 3-5 days per week including aerobic and muscle strengthening exercises.

›› As you progress, you should aim to accumulate 150 minutes or more of moderate-intensity physical activity per week.

›› If you are over the age of 45 yrs. and NOT accustomed to regular vigorous physical activity, please consult a qualified exercise professional (CSEP-CEP) before engaging in maximal effort exercise.

If you answered YES to one or more of the follow-up questions about your medical condition:

›› You should seek further information from a licensed health care professional before becoming more physically active or engaging in a fitness appraisal and/or visit a or qualified exercise professional (CSEP-CEP) for further information.

!

Delay becoming more active if:

›› You are not feeling well because of a temporary illness such as a cold or fever – wait until you feel better

›› You are pregnant - talk to your health care practitioner, your physician, a qualified exercise profesional, and/or complete the PARmed-X for Pregnancy before becoming more physically active OR

›› Your health changes - please talk to your doctor or qualified exercise professional (CSEP-CEP) before continuing with any physical activity programme.

SECTION 3 - DECLARATION

›› You are encouraged to photocopy the PAR-Q+. You must use the entire questionnaire and NO changes are permitted.

›› If you are less than the legal age required for consent or require the assent of a care provider, your parent, guardian or care provider must also sign this form.

›› Please read and sign the declaration below:

I, the undersigned, have read, understood to my full satisfaction and completed this questionnaire. I acknowledge that this physical activity clearance is valid for a maximum of 12 months from the date it is completed and becomes invalid if my condition changes. I also acknowledge that a Trustee (such as my employer, community/fitness centre, health care provider, or other designate) may retain a copy of this form for their records. In these instances, the Trustee will be required to adhere to local, national, and international guidelines regarding the storage of personal health information ensuring that they maintain the privacy of the information and do not misuse or wrongfully disclose such information.

NAME ____ DATE ____

SIGNATURE _____________________________________WITNESS ____

SIGNATURE OF PARENT/GUARDIAN/CARE PROVIDER ____

For more information, please contact: Canadian Society for Exercise Physiology www.csep.ca KEY

REFERENCES:

1. Jamnik VJ, Warburton DER, Makarski J, McKenzie DC, Shephard RJ, Stone J, and Gledhill N. Enhancing the eectiveness of clearance for physical activity participation; background and overall process. APNM 36(S1):S3- S13, 2011.

2. Warburton DER, Gledhill N, Jamnik VK, Bredin SSD, McKenzie DC, Stone J, Charlesworth S, and Shephard RJ. Evidence-based risk assessment and recommendations for physical activity clearance; Consensus Document. APNM 36(S1):S266-s298, 2011.
